# Effects of micro- and macro-stressors and resilience factors on the mental health of parents caring for chronically ill and disabled children and adolescents

**DOI:** 10.1186/s12912-025-03125-6

**Published:** 2025-05-02

**Authors:** Jan Broll, Sarah K. Schäfer, Daniel Lüdecke, Stefan Nickel, Klaus Lieb, Isabella Helmreich

**Affiliations:** 1 Leibniz Institute for Resilience Research (LIR), Wallstraße 7, D-55122 Mainz, Germany; 2 Clinical Psychology, Psychotherapy and Psychodiagnostics, Technical University of Braunschweig, D-38106 Braunschweig, Germany; 3 Institute of Medical Sociology, University Medical Center Hamburg-Eppendorf, D-20246 Hamburg, Germany

**Keywords:** Resilience, Mental health, Protective factors, Parents, Child, Disabilities

## Abstract

**Background:**

This study examines the impact of non-care-related stressors and resilience factors on the mental health of caregivers for chronically ill and disabled children. It aims to identify the daily stressors and protective factors most relevant to caregiver well-being.

**Methods:**

A total of 202 caregivers (predominantly female, aged 25–59) participated in a survey measuring exposure to daily micro-stressors, such as household tasks and financial pressures, and macro-stressors, such as significant life events. Resilience factors, including social support and internal locus of control, were also assessed. Descriptive statistics and regression analyses explored the relationship between stressors, resilience factors, and psychological distress.

**Results:**

Micro-stressors were strongly associated with higher levels of psychological distress, underscoring the cumulative burden of frequent, daily stressors. In contrast, macro-stressors had no significant impact on distress, possibly due to their lower frequency or differences in perception. Resilience factors, particularly social support and internal locus of control, buffered against distress, demonstrating their protective role. Internal locus of control moderated the relationship between micro-stressors and distress, indicating that caregivers who felt greater personal control over their circumstances were better able to manage the negative effects of daily stressors.

**Conclusions:**

The findings highlight the significant toll of daily micro-stressors on caregivers’ mental health and emphasize the important role of resilience factors in mitigating this burden. Strengthening caregivers’ social networks and fostering internal control beliefs could be key components of interventions designed to improve their well-being. These results suggest that supporting caregivers is essential not only to enhance their quality of life but also to sustain their caregiving roles. Further research should investigate the long-term effects of interventions targeting resilience and explore additional protective factors that may buffer against daily stressors in this vulnerable population. These findings have important implications for disability and rehabilitation services aiming to provide holistic caregiver support.

**Trial registration:**

DRKS00027465, 2022-01-04 (German Clinical Trials Register); NCT05418205, 2022-03-01 (ClinicalTrials.gov).

## Introduction

Chronically ill and disabled children are individuals who experience ongoing health conditions that require continuous case management and intervention [1], usually because they have at least one of the following functional impairments: need for medication, medical attention, and/or modified schooling [2]. Most of these children are cared for by their parents at home [3]. These parents often face unique challenges and significant stress related to their caregiver role [3, 4]. These challenges include financial, emotional and physical burdens [5], increasing the risk of health, social and economic problems, often leading to poorer living conditions and care situations [6]. In addition to stressors related to their caregiver role, these parents are exposed to similar stressors as parents of children without disabilities such as household responsibilities, balancing work and family life, and navigating everyday challenges like transportation and childcare [7–9]. In contrast to parents of children living without disabilities, little is known about the stressors relevant for parents of children with chronic health conditions.

Stressors represent stimuli or situations that elicits stress responses [10]. A common way to quantify stressors is to distinguish between micro- and macro-stressors [11]. Micro-stressors are daily hassles, that is, small everyday stressors that individuals experience frequently such as time pressure at work or missing a bus [12]. These stressors are often minor annoyances, irritations, or challenges that can accumulate over time and contribute to overall stress levels [13]. They can have profound negative effects on mental health and well-being, especially when they are numerous and persistent [14, 15].

Macro-stressors, on the other hand, are larger, more significant sources of stress that have a broader impact on an individual’s life [12]. These stressors typically result from major life events, transitions, or persistent difficult circumstances. Examples of macro-stressors include divorce, job loss, major financial problems, chronic illness, major accidents, and natural disasters. Unlike micro-stressors, macro-stressors tend to have a more immediate and serious effect on an individual’s stress levels and may require more coping behavior to be managed successfully [16].

Parents of chronically ill and disabled children are exposed to a variety of stressors. Reviews showed that these parents are exposed to greater parenting stress than parents of children without major health challenges [17–19]. Increased parenting stress may result from heightened parental accountability in managing treatments and was found to be influenced by the duration and severity of children’s illnesses [17]. Moreover, parents of chronically ill and disabled children and adolescents also need to invest more in coping strategies to manage increased stressor exposure [20].

Few studies in this field have addressed stressors unrelated to the child’s illness. Many parents report that the stress of caring for their child leaves them unable to cope with everyday difficulties [21]. They often feel overwhelmed, exhausted, and stressed [22]. Increased stress associated with caring can spill over to other areas of life [23], because the demands of caregiving consume resources that would otherwise be used to fulfil other social roles [24]. For example, the work-life balance of caregivers tends to be imbalanced [25]. Kish et al. [26] reviewed stressors affecting parents of children with chronic illness, identifying three main sources of stress: workplace, family/child, and personal challenges. Workplace-related stress stemmed largely from inflexibility, while family-related stress involved finding suitable childcare, time constraints, and the pressure to support both the disabled child and the family. Personal challenges included a lack of optimism and uncertain perspectives of the future. Together, these stressors contributed to a reduced quality of life for parents of chronically ill children and adolescents.

When people maintain or quickly recover their mental health in face of such stressors, they respond resiliently to those stressors [27, 28]. A growing body of research has demonstrated a positive association between resilience factors and resilient outcomes (i.e., low levels of mental distress or high levels of well-being) in the general population [29]. This association is also found in parents of chronically ill children [30]. Research shows that certain resilience factors (such as social support and optimism) help people to cope with stressful life events and mitigate risk factors [31–33].

## Aims and objective

This study aimed to (1) identify the stressors faced by parents of chronically ill and disabled children and adolescents, encompassing both micro- and macro-stressors; (2) investigate the differential influence of micro- and macro-stressors on mental health outcomes in those parents; and (3) identify potential resilience factors that may have the potential to alleviate the impact of stressors on mental health.

## Method

### Study design and sample recruitment

The data used in this study were collected as part of the NEST research project [34] in Germany in February and March 2022. The NEST project aims to evaluate a case management intervention for families with chronically ill and disabled children and adolescents. While the overall project follows a longitudinal design, the present analysis is based solely on baseline data, making it a cross-sectional study. Participants completed the baseline survey via the online platform SoSci Survey [35], which facilitated data collection. Participants engaged in the study through the online platform SoSci Survey [35], which facilitates data collection.

A total of 202 participants were recruited from two established German support networks for parents of children in need of care, nestwärme e. V. and Kindernetzwerk e. V. The sample size planning for the NEST project was based on the planned intervention; however, no separate sample size planning was conducted for the specific analyses presented in this paper. To be eligible for inclusion in the study, participants had to meet two inclusion criteria: (1) to be a parent of a child with a disability and/or chronic disease according to the criteria of the German Social Code (receipt of benefits according to § 37 SGB V and/or care level > 1), and (2) to be at least 18 years old. For a comprehensive description of the inclusion and exclusion criteria, see Nickel et al. [34]. The study was approved by the local ethics committee of the University Medical Center Hamburg-Eppendorf (application number: LPEK-0370). All subjects gave informed consent in accordance with the Declaration of Helsinki and its latest revisions. While the project itself was prospectively registered on the German Clinical Trials Register (ID: DRKS00027465) as well as on ClinicalTrials.gov (ID: NCT05418205), the specific analyses performed in this study were not part of the registration.

### Materials

The online survey included the following sections: sociodemographic data, access to and use of utilities, mental health, resilience and resilience-related constructs, stressor exposure (see Nickel et al., 2023, for details). All instruments used in this study were previously validated and published. No new instruments were developed specifically for this study. Below, we provide a description of the validated tools used in the analysis. In this paper, we focus on the following outcomes and risk/resilience factors; other results will be published elsewhere.

#### Mainz Inventory of Microstressors (MIMIS)

Micro-stressors were assessed using the MIMIS [36]. The MIMIS provides a retrospective assessment of micro-stressors over a one-week period. The questionnaire consists of 58 items that assess micro-stressor load over the past seven days. It separates stressor occurrence from perceived stressor severity. The occurrence of stressors is measured on an 8-point scale (0–7; 0 = did not occur, 7 = occurred on seven days). For each participant, a total stressor score was calculated by summing the frequency of all reported stressors, resulting in an overall measure of stressor. This total score ranged from 0 to 406. The perceived severity of the stressor is rated on a four-point scale (0–4; 0 = not at all severe, 4 = extremely severe). A total severity score was calculated by summing the severity ratings for all reported stressors. This total severity score ranged from 0 to 232. In the current study, the internal consistency was good, ω = 0.89, 95% CI [0.88; 0.93].

#### Life Events (LE)

Macro-stressors were assessed using the checklist for life events developed by Canli et al. [37] which contains 27 events. Respondents are asked to indicate whether a particular event occurred within a specified period, in our case, the last 6 months. For each participant, an overall score was calculated based on the total number of events experienced during this period. Since the number of potential events was not fixed, the overall score was theoretically open-ended, depending entirely on how many stressors were reported. Respondents were then asked to indicate on a 5-point scale how severe the respective event was (1–5; 1 = not at all severe, 5 = extremely severe). An overall severity score was calculated by summing the severity ratings for all events that occurred. This overall score was dependent on the total number of events experienced, and because the number of potential events was not fixed, the score was also theoretically open-ended. In the current study, internal consistency was acceptable, ω = 0.70, 95% CI [0.68; 0.72].

#### General Health Questionnaire-28 (GHQ-28)

Mental distress was measured using the 28-item version of the General Health Questionnaire [38]. The GHQ-28 assesses somatic symptoms, anxiety and insomnia, social dysfunction, and severe depression with 28 items on a 3-point scale (0–3; 0 = better than usual, 3 = much worse than usual). Higher scores indicate more severe mental distress. In the present study, internal consistency was excellent, ω = 0.93, 95% CI [0.91; 0.94].

#### Perceived Stress Scale 4 (PSS-4)

Perceived stress was measured using the PSS-4 [39], with 4 items on a 5-point scale (0–4; 0 = never, 4 = very often). Higher scores indicate more severe subjectively perceived stress. In the current study, internal consistency was acceptable, ω = 0.77, 95% CI [0.71; 0.82].

#### Optimism Pessimism Scale 2 (SOP-2)

Parents’ dispositional optimism and pessimism was measured using the SOP-2 [40]. The two items are rated on a 7-point scale (1–7; 1 = not at all optimistic/pessimistic, 7 = very optimistic/pessimistic). Higher scores indicate more dispositional optimism. Internal consistency was good in our sample, ω = 0.89, 95% CI [0.85; 0.93].

#### General Self-Efficacy short scale-3 (GSE–3)

Parents’ perceived self-efficacy beliefs were measured using the GSE–3 [41]. Three items are rated on a 5-point scale (1–5; 1 = does not apply at all, 5 = applies completely). Higher scores indicate stronger self-efficacy beliefs. In our sample, the internal consistency of the measure was good, *ω* = 0.84, 95% CI [0.83; 0.85].

#### Oslo Social Support Scale (OSSS-3)

Perceived social support was measured using the OSSS-3 [42], which consists of 3 items that are rated on a 4-5-point scale (Item 1: 1 = none, 5 = more than 5; Item 2: 1 = none, 5 = a lot, and for Item 3: 1 = very difficult, 5 = very easy). Higher scores indicate greater perceived social support. In the current study, the internal consistency was acceptable, *ω* = 0.65, 95% CI [0.57; 0.73].

#### Internal-External locus of control short scale–4 (IE-4)

Internal locus of control was measured using the internal locus of control subscale of the Internal-External Locus of Control Short Scale-4 [43], with two items rated on a 5-point scale (1–5; 1 = does not apply at all, 5 = applies completely). Higher scores indicate greater internal locus of control. In the current study, the internal consistency was acceptable, *ω* = 0.70, 95% CI [0.59; 0.78].

#### Resilience Scale for Adults (RSA)

We used the family cohesion subscale of the Resilience Scale for Adults [44] to measure family cohesion. This subscale, which can also be used as a stand-alone scale, consists of 6 items, rated on a 6-point scale (1–6), with varying item-specific anchors. Higher scores indicate greater family cohesion. In the current study, the internal consistency was acceptable, *ω* = 0.74, 95% CI [0.60; 0.80].

### Data analyses

Analyses were conducted using *R* version 4.3.2 [45] and the packages *easystats* [46], and *tidyverse* [47].

Descriptive analyses, including means, standard deviations, and frequencies, were used to characterize the sample demographics and caregiving context. Additionally, descriptive statistics were employed to explore the prevalence and perceived severity of both common and most severe micro- (daily hassles)- and macro-stressors (life events). Furthermore, heat maps illustrating the frequency and severity of micro-stressors were generated, providing a visual representation of patterns through varying color intensities.

To better understand the relationship between stress and mental health, we employed linear regression models. Each model examined different aspects of stressors, resilience factors, and mental health outcomes. With respect to those models, we use the term predictor for independent variables in regression analyses, which does not necessarily imply a causal relationship. For regression analyses, we used linear models adjusted for age, gender, education, care level and income, which were included as control variables to account for potential confounding effects. Age and income were treated as continuous variables, while gender, care level and education were coded as categorical variables. The models were structured as follows:

- Model 1: The outcome was mental distress, with the sum of micro-and macro-stressors as predictors.
- Model 2: The outcome was mental distress, predictors included severity of micro- and macro-stressors.
- Model 3: We examined the interaction between mental distress, sum of micro- and macro-stressors, and resilience factors. The resilience factors self-efficacy, social support, optimism, internal locus of control, and family cohesion served as moderators of the relationship between stressor exposure and mental distress in this analysis.

The original power analysis was based on a multilevel model to account for potential regional variation in caregiver experiences. Assuming 5% intra-cluster variance and using a variance inflation factor [48], the required sample size was estimated at 73 participants per group. Accounting for 20–40% expected attrition [49] the target sample size was set at *N* = 202. For the present analysis, only baseline data were used. Post hoc power analyses were conducted for the three multiple regression models using observed effect sizes: Cohen’s *f*^2^ [50]. Model 1 (*f*^2^ = 0.23, df = 174) showed a power of 0.98; model 2 (*f*^2^ = 0.28, df = 180) a power of 0.99; and model 3 (*f*^2^ = 0.72, df = 159) a power of 0.97. All models had sufficient power (> 0.80) to detect the observed effects.

## Results

### Sample characteristics

The sample consisted of 202 caregivers (see Table 1 for sociodemographic characteristics with a mean age of 42 years (*SD* = 6.75), ranging from 25 to 59 years. The majority of the parents were female (93%), with only 7% being male. In terms of marital status, 77% were in a relationship, while 23% were either single or divorced. Regarding educational background, 36% held a university degree, 29% completed high school, 22% had a medium secondary education, and 6% completed low secondary education, with only 0.52% having no school-leaving qualification.

**Table 1:** Descriptive characteristics of parents and children in the sample (*N* = 202)

Parents	
**Age (years)**	
Mean (SD)	41.63 (*6.75*)
Median (Min; Max)	41.00 (*25; 59*)
**Sex (frequency**,** %)**	
Female	187 (92.57%)
Male	15 (7.43%)
**Marriage status (frequency**,** %)**	
Single	23 (11.39%)
Relationship	156 (77.22%)
Divorced	23 (11.39%)
**Education (frequency**,** %)**	
No school-leaving qualification	1 (0.52%)
Low secondary education	13 (6.40%)
Medium secondary education	45 (22.17%)
High school	58 (28.57%)
University degree	70 (36.46%)
Other	5 (2.46%)
***Children***	
**Age (years)**	
Mean (SD)	7.58 (*4.35*)
Median (Min; Max)	7 (*1; 17*)
**Sex (frequency**,** %)**	
Female	90 (44.55%)
Male	112 (55.45%)
**Care level (frequency**,** %)**	
1	7 (3.66%)
2	25 (13.09%)
3	60 (31.41%)
4	56 (29.32%)
5	43 (22.51%)

*Note. n*, number of cases; *SD*, standard deviation; *Min*, minimum; *Max*, maximum

The children in the sample had a mean age of 8 years (*SD* = 4.35), with a median age of 7, ranging from 1 to 17 years. Slightly more children were male (55%) than female (45%). Germany has a system for assessing the severity of illnesses through care levels (“Pflegegrade”), which categorize an individual’s need for care and determine their access to support services and financial assistance [51]. Care levels range from 1 (lowest) to 5 (highest), with level 1 representing a mild need for care and level 5 indicating severe dependence on assistance for daily activities. The care levels of the children in our sample varied, with the majority having a care level of 3 (31%) or 4 (29%), while smaller proportions were classified as level 5 (23%), level 2 (13%), and level 1 (4%). The children in the sample had a wide range of chronic illnesses and disabilities, including genetic syndromes (e.g., Down syndrome, Rett syndrome, Prader-Willi syndrome, Costello syndrome), neurodevelopmental disorders (e.g., autism spectrum disorder, cerebral palsy, developmental delays), metabolic and muscular diseases (e.g., mitochondrial disorders, Duchenne muscular dystrophy, glycogen storage disease), and severe congenital conditions (e.g., spina bifida, heart defects, epilepsy). Many children experienced co-occurring conditions, such as intellectual disabilities, motor impairments, and feeding or sensory difficulties.

### Stressor load

We analyzed both micro- and macro-stressors, focusing on their frequency and severity. The most common micro-stressor was household chores, reported by 95% of participants, followed by lack of sleep (89.6%), interruptions of activities (86%), negative events in the media (86%), and negative political events (85%). Regarding severity, money problems (mean severity rating of 3.92, experienced by 27.72% of participants), lack of support (3.90, 66.34%), time pressure (3.89, 24.75%), and lack of sleep (3.87, 89.60%) were identified as the most burdensome micro-stressors. The most prevalent macro-stressor was serious illness or accident of close relative (96%), followed by selling or moving house (67%), death of a close relative (59%), wedding plans (54%) and child’s first day at school (50%). The most burdensome macro-stressors were victim of physical abuse (mean severity rating of 5.00, experienced by 0.50% of participants), death of a beloved pet (4.50, 0.99%), separation of parents (4.33, 1.49%), pregnancy complications or miscarriage (4.33, 0.99%), and serious illness or accident (4.31, 0.50%).

Table 2 presents the total values of micro and macro stressors (sum and severity scale).

**Table 2:** Mean and standard deviation of micro- and macro-stressors in intervention and control groups

Stressor type	
**Micro-stressors**	
Sum	92.88 (43.78)
Severity	85.90 (37.25)
**Macro-stressors**	
Sum	8.64 (4.44)
Severity	34.28 (24.42)

Note. Values are presented as mean (standard deviation)

**Fig. 1:**
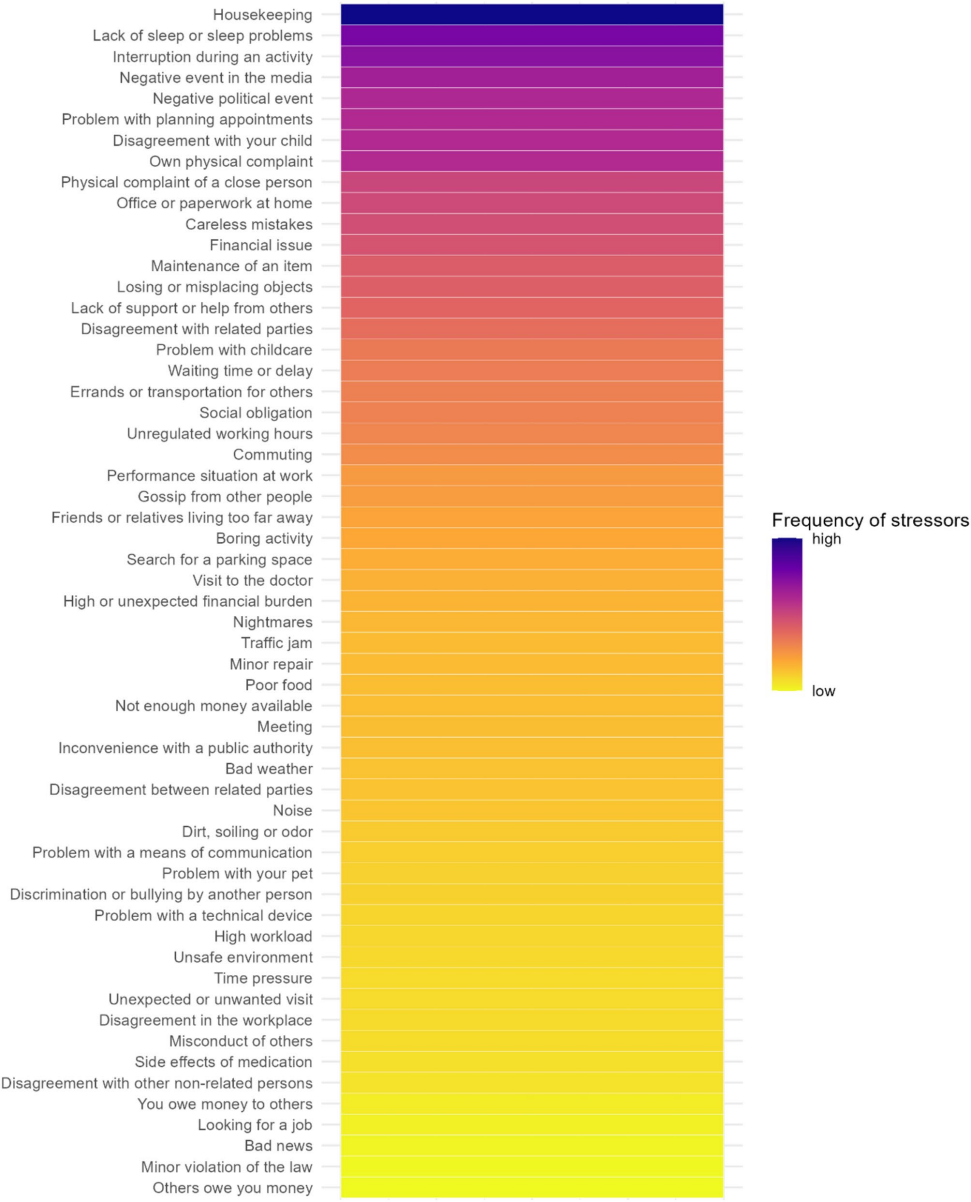
Heat map of frequency of micro-stressors

**Fig. 2:**
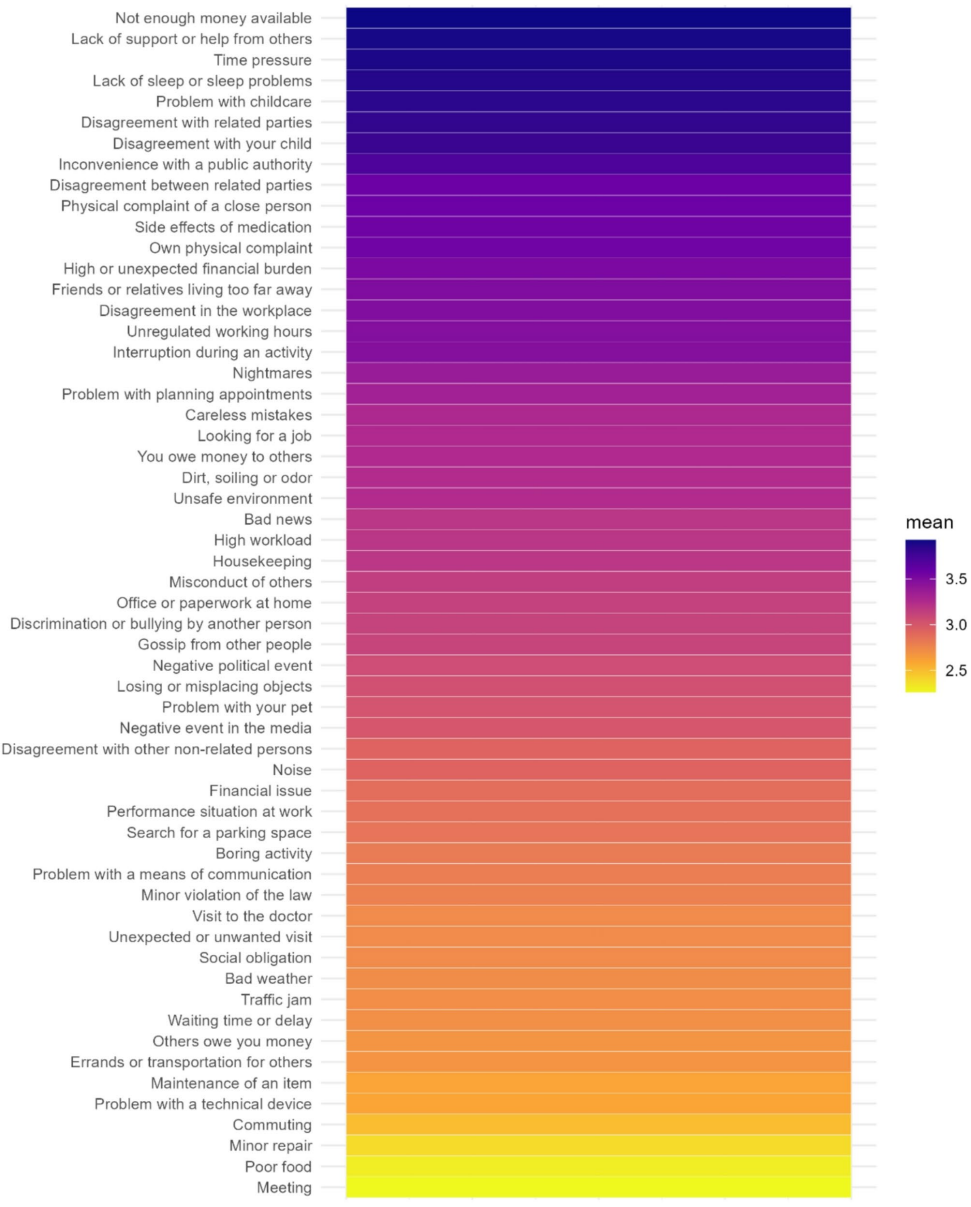
Heat map of severity of micro-stressors

For a clear presentation of the micro-stressors, Figs. 1 and 2 display heat maps representing the **frequency** (count) and **severity** (mean value) of these stressors. The frequency heat map illustrates how often each stressor occurred among participants, while the severity heat map highlights the average perceived intensity of each stressor. These visualizations help identify patterns of stressor exposure, highlighting which types of daily challenges were most commonly experienced and perceived as most intense.

### Stressor load and mental health

The results of regression model 1 are presented in Table 3.

**Table 3:** Regression analysis of the impact of sum of micro- and macro-stressors, demographic factors, and income on mental distress

Parameter	*b*	95% CI	*t*	*p*	b* ^*1*^	95% CI (b*)	Fit
Intercept	54.12	[37.74, 70.50]	6.52	< 0.001	-	-	
Sum of Micro-Stressors	0.10	[0.06, 0.15]	4.40	< 0.001	0.33	[0.18, 0.47]	
Sum of Macro-Stressors	0.20	[-0.23, 0.62]	0.92	0.361	0.07	[-0.08, 0.21]	
Age	0.06	[-0.22, 0.33]	0.41	0.686	0.03	[-0.11, 0.17]	
Gender (male)	-7.46	[-14.38, -0.53]	-2.12	0.055	-0.58	[-0.11, 0.17]	
Education Level (middle)	1.84	[-5.25, 8.93]	0.51	0.609	0.14	[-0.11, 0.04]	
Education Level (high)	2.10	[-4.41, 8.60]	0.64	0.525	0.16	[-0.34, 0.66]	
Income	-1.07	[-1.95, -0.18]	-2.38	0.018	-0.17	[-0.32, -0.03]	
Care level (2)	0.36	[-9.93, 10.65]	0.07	0.945	0.03	[-0.77, 0.82]	
Care level (3)	-0.23	[-9.84, 9.37]	-0.05	0.962	-0.02	[-0.76, 0.72]	
Care level (4)	0.71	[-8.91, 10.33]	0.15	0.884	0.06	[-0.69, 0.80]	
Care level (5)	1.23	[-8.58, 11.03]	0.25	0.806	0.09	[-0.66, 0.85]	
AIC							1,467.05
BIC							1,508.98
R^2^							0.19
R^2^ (adj.)							0.13

*Note: b =* unstandardized coefficient; *df* = degrees of freedom, b* = Standardized coefficient; *CI* = Confidence Interval; *AIC* = Akaike information criterion; *BIC* = Bayesian information criterion; ^*1*^ All predictors were z-standardized (*M* = 0, *SD* = 1); the outcome variable remained unstandardized and is presented in its normal units of measurement. The intercept reflects the expected value of the outcome when all predictors are set to their mean (z = 0)

The results showed that the frequency of micro-stressors was significantly positively associated with mental distress (*p* < 0.001). Individuals with higher micro-stress exposure tended to have higher psychological distress. In contrast, the frequency of macro-stressors was not significantly associated with mental distress (*p* = 0.149). The model explained approximately 18% of the variance in mental distress (*R*^2^ = 0.181; adjusted *R*^2^ = 0.134).

The results of regression model 2 are presented in Table 4.

**Table 4:** Regression analysis of the impact of the severity of micro- and macro-stressors, demographic factors, and income on mental distress

Parameter	*b*	95% CI	*t*	*p*	b* ^*1*^	95% CI (b*)	Fit
Intercept	52.28	[36.45, 68.11]	6.52	< 0.001	-0.20	[-1.04, 0.64]	
Severity of Micro-Stressors	0.14	[0.09, 0.19]	5.51	< 0.001	0.41	[0.26, 0.55]	
Severity of Macro-Stressors	0.00	[-0.07, 0.08]	0.07	0.946	0.00	[-0.14, 0.15]	
Age	0.04	[-0.22, 0.30]	0.33	0.742	0.02	[-0.11, 0.16]	
Gender (male)	-6.77	[-13.51, -0.03]	-1.98	0.049	-0.53	[-1.05, 0.00]	
Education Level (middle)	2.33	[-4.46, 9.12]	0.68	0.499	0.18	[-0.35, 0.71]	
Education Level (high)	2.56	[-3.73, 8.85]	0.80	0.423	0.20	[-0.29, 0.69]	
Income	-0.94	[-1.79, -0.08]	-2.16	0.032	-0.15	[-0.29, -0.01]	
Care level (2)	0.71	[-9.21, 10.64]	0.14	0.888	0.06	[-0.72, 0.83)	
Care level (3)	1.03	[-8.29, 10.34]	0.22	0.828	0.08	[-0.64, 0.80]	
Care level (4)	0.72	[-8.58, 10.02]	0.15	0.879	0.06	[-0.67, 0.78]	
Care level (5)	0.74	[-8.75, 10.24]	0.15	0.877	0.06	[-0.68, 0.80]	
AIC							1,502.51
BIC							1,544.86
R^2^							0.22
R^2^ (adj.)							0.17

*Note: b =* unstandardized coefficient; *df* = degrees of freedom, b* = Standardized coefficient; *CI* = Confidence Interval; *AIC* = Akaike information criterion; *BIC* = Bayesian information criterion; ^*1*^ All predictors were z-standardized (*M* = 0, *SD* = 1); the outcome variable remained unstandardized and is presented in its normal units of measurement. The intercept reflects the expected value of the outcome when all predictors are set to their mean (z = 0)

The results showed that the perceived severity of micro-stressors was significantly positively associated with mental distress (*p* < 0.001), indicating that individuals who perceived micro-stressors as more severe experienced higher levels of mental distress. By contrast, the perceived severity of macro-stressors was not significantly associated with mental distress (*p* = 0.435). The model accounted for approximately 22% of the variance in mental distress (*R*^2^ = 0.223; adjusted *R*^2^ = 0.171).

### Resilience factors

Our next aim was to examine the moderator effect of resilience factors on the relationship between stressor exposure (including micro- and macro-stressors) and mental distress. The results of the regression analysis (model 3) are presented in Table 5.

**Table 5:** Regression analysis for the association between sum of micro- and macro-stressors, resilience factors, and mental distress

Parameter	*b*	95% CI	*t*	*p*	b* ^*1*^		95% CI (b*)	Fit
Intercept	92.24	[59.17, 125.31]	5.51	< 0.001	-0.25		[-1.06, 0.56]	
Sum of Micro-Stressors	-0.08	[-0.36, 0.20]	-0.57	0.572	0.25		[0.11, 0.39]	
Sum of Macro-Stressors	0.54	[-2.20, 3.27]	0.39	0.699	0.01		[-0.12, 0.15)	
Self-Efficacy	-6.78	[-15.50, 1.93]	-1.54	0.005	-0.10		[-0.27, 0.08]	
Optimism	-1.63	[-5.68, 2.43]	-2.87	0.430	-0.20		[-0.36, -0.05]	
Social Support	-3.70	[-6.26, -1.15]	-0.79	0.004	-0.08		[-0.23, 0.08]	
Internal Control	-3.28	[-5.47, -1.09	1.11	0.001	-0.03		[-0.20, 0.14]	
Family Cohesion	-0.97	[-2.66, 0.72]	-1.14	0.258	-0.17		[-0.32, -0.02]	
Age	0.13	[-0.13, 0.38]	0.98	0.327	0.07		[-0.07, 0.20]	
Gender (male)	-4.25	[-10.61, 2.12]	-1.32	0.190	-0.33		[-0.82, 0.16]	
Education Level (middle)	2.37	[-4.12, 8.86]	0.72	0.471	0.18		[-0.32, 0.69]	
Education Level (high)	2.24	[-3.84, 8.31]	0.73	0.468	0.17		[-0.30, 0.64]	
Income	-0.62	[-1.46, 0.21]	-1.47	0.143	-0.10		[-0.24, 0.03]	
Care level (2)	2.62	[-7.38, 10.86]	0.54	0.591	0.20		[-0.54, 0.95]	
Care level (3)	2.33	(-7.00, 12.23]	0.51	0.610	0.18		[-0.52, 0.88]	
Care level (4)	2.80	[-6.69, 11.36]	0.62	0.536	0.22		(-0.47, 0.90]	
Care level (5)	1.74	[-6.10, 11.69]	0.38	0.707	0.13		(-0.57, 0.84]	
Sum of Micro-Stressors x Self-Efficacy	0.08	[-0.02, 0.17]	1.65	0.101	0.18		[-0.04, 0.39]	
Sum of Micro-Stressors x Optimism	0.006	[-0.03, 0.04]	0.36	0.722	0.03		[-0.14, 0.21]	
Sum of Micro-Stressors x Social Support	0.02	[0.001, 0.04]	2.08	0.039	0.15		[0.007, 0.29]	
Sum of Micro-Stressors x Internal Control	-0.08	[-0.15, -0.02]	-2.48	0.014	-0.23		[-0.41, -0.05]	
Sum of Micro-Stressors x Family Cohesion	−0.002	[-0.02, 0.01]	-0.39	0.694	-0.03		[-0.19, 0.12]	
Sum of Macro-Stressors x Self-Efficacy	-0.25	[-1.05, 0.54]	-0.63	0.531	-0.06		[-0.26, 0.13]	
Sum of Macro-Stressors x Optimism	-0.09	[-0.45, 0.26]	-0.52	0.601	-0.05		[-0.22, 0.13]	
Sum of Macro-Stressors x Social Support	0.14	[-0.08, 0.36]	1.25	0.214	0.10		[-0.06, 0.27]	
Sum of Macro-Stressors x Internal Control	-0.35	[-0.96, 0.25]	-1.16	0.249	-0.10		[-0.27, 0.07]	
Sum of Macro-Stressors x Family Cohesion	0.07	[-0.08, 0.21]	0.92	0.360	0.08		[-0.09, 0.24]	
AIC								1,434.53
BIC								1,524.85
R^2^							0.42	
R^2^ (adj.)							0.32	

*Note: b =* unstandardized coefficient; *df* = degrees of freedom, b* = Standardized coefficient; *CI* = Confidence Interval; *AIC* = Akaike information criterion; *BIC* = Bayesian information criterion; ^*1*^All predictors were z-standardized (*M* = 0, *SD* = 1); the outcome variable remained unstandardized and is presented in its normal units of measurement. The intercept reflects the expected value of the outcome when all predictors are set to their mean (z = 0)

The regression analysis revealed significant relationships between certain resilience factors, stressor interactions, and psychological distress. Micro-stressors alone were not significantly associated with psychological distress (*p* = 0.572). However, resilience factors played a critical role: internal locus of control (*p* = 0.001) and social support (*p* = 0.005) were significantly associated with reduced psychological distress.

Interaction effects showed that internal locus of control moderated the relationship between micro-stressors and distress, buffering against their negative impact (*p* = 0.014). Similarly, social support interacted with micro-stressors to reduce their influence on distress (*p* = 0.039). Macro-stressors and their interactions with resilience factors did not significantly predict distress (*p* > 0.05).

## Discussion

The current study offered a more profound understanding of micro- and macro-level stressors and their correlation with mental health, along with identifying protective factors within a sample of parents caring for chronically ill and disabled children.

The analysis of micro- and macro-stressors among parents of chronically ill children revealed a range of common and severe stressors. Household chores, lack of sleep, and financial issues were the most frequently reported micro-stressors, with money problems and lack of support being rated as the most severe. In terms of macro-stressors, serious illness or accidents and moving house were most frequently encountered, while rather low frequent events like physical abuse and the death of a pet were perceived as the most burdensome. Given the predominance of women in our sample, this finding aligns with previous research on gender inequalities in domestic work [52]. The findings underscore the significant double burden faced by parents of chronically ill children, as they navigate both the demands of caregiving and the strain of everyday stressors. This dual challenge is particularly troubling because micro-stressors like lack of sleep and financial difficulties are frequent and on-going. Understanding this double burden is critical when designing support systems for parents. It highlights the need for interventions that target both aspects of their stress. On one level, support must be provided for managing the acute crises related to their child’s illness (e.g., medical emergencies, treatment management). At the same time, efforts must also be made to alleviate the constant pressure of everyday life. For instance, providing financial aid, respite care, or support with household tasks could significantly reduce the weight of micro-stressors, allowing parents more time and energy to focus on their child’s care without feeling overwhelmed by the necessities of daily life. Additionally, mental health interventions should consider how chronic stress from micro-stressors might accumulate and intensify parents’ vulnerability [53, 54].

Our regression analyses showed that both the number and perceived severity of stressors were significantly linked to psychological distress. However, micro-stressors had a greater influence on distress levels than macro-stressors, reinforcing that daily, minor stressors can have a substantial impact on mental health [26]. Individuals experiencing a higher number of micro-stressors or perceiving them as more severe reported increased psychological distress. Although macro-stressors were also associated with distress, their impact was less pronounced. These models explained a substantial portion of the variance in mental health outcomes, highlighting the significant role daily micro-stressors play in overall well-being. This finding is consistent with other studies which have also found that exposure to micro-stressors can contribute significantly to the deterioration of short term and ultimately long term affective well-being [55–57]. and even exceeded the relevance of macro-stressors like major life events [56, 58–60].

Our study aimed to elucidate the role of protective factors, specifically resilience factors, in mitigating the impact of stressors on caregiver psychological distress. Our analysis also confirmed that resilience factors can have a protective effect against psychological distress for caregivers [30, 61]. Notably, social support and family cohesion emerged as important protective factors, which is in line with previous studies [18, 62, 63]. Additionally, the significant interaction between micro-stressors and internal control suggests that individuals with a greater sense of internal control may be better equipped to cope with increasing micro-stressors, thereby mitigating their negative effects. These findings highlight the importance of enhancing internal control and strengthening social support networks to buffer against the adverse effects of daily stressors [18, 62, 63]. In contrast, macro-stressors and other variables, such as age, gender, education, and income, did not show significant associations, suggesting that the cumulative burden of micro-stressors may play a more crucial role in influencing mental distress than these broader factors.

### Strengths and limitations

This study provided a comprehensive evaluation of micro- and macro-stressors, offering a detailed understanding of the stressors impacting caregivers of chronically ill children. By examining resilience factors such as social support, optimism, internal locus of control, and family cohesion, the study contributed to a deeper understanding of protective mechanisms against psychological distress.

However, our study must be interpreted in the light of its limitations. First, our sample is not representative for the population of parents with children in need of care in Germany. The sample of the current study was recruited via specific support organizations. While this approach allowed us to access a population of interest, it is essential to acknowledge the potential presence of selection bias within our sample. It is plausible that families in our sample have higher levels of awareness and access to available support services and experience more social support [64, 65]. Second, reliance on self-reported data introduced potential biases affecting the accuracy of reported stress and distress levels. Additionally, other relevant variables influencing the stress-distress relationship, such as cultural differences and individual coping strategies, were not accounted for in this study. Third, the gender distribution in our sample was highly imbalanced, with 93% of participants identifying as women. This overrepresentation of mothers is common in caregiving research, as women continue to take on the majority of care responsibilities in families [66]. Although our research focuses on parents, this skewed distribution limits the representativeness of the findings, particularly with regard to fathers and caregivers self-identifying as men. Consequently, interpretations of stress exposure and resilience may be more reflective of maternal experiences, which should be taken into account when generalizing the results to the broader population of parents.

## Conclusion

This study provides a deeper understanding of the impact of micro- and macro-stressors on the mental health of parents caring for chronically ill and disabled children, emphasizing the critical role of daily stressors. Macro-stressors, such as serious illness in family members, also contribute to distress, though their impact is less pronounced compared to micro-stressors.

The results may be of relevance for the development of targeted interventions that address both caregiving demands and everyday micro-stressors to improve overall well-being. Moreover, the study highlights the protective effects of resilience factors, with social support, family cohesion, and a strong sense of internal control emerging as crucial buffers against daily stressors. This suggests that promoting resilience and strengthening support networks may be more effective in enhancing caregiver mental health than merely reducing stressors. This, however, has to be proved in further studies.

## Data Availability

The datasets used and/or analysed during the current study are available from the corresponding author on reasonable request.
